# Phosphorylation-Dependent 14-3-3 Binding to LRRK2 Is Impaired by Common Mutations of Familial Parkinson's Disease

**DOI:** 10.1371/journal.pone.0017153

**Published:** 2011-03-01

**Authors:** Xianting Li, Qing Jun Wang, Nina Pan, Sangkyu Lee, Yingming Zhao, Brian T. Chait, Zhenyu Yue

**Affiliations:** 1 Department of Neurology and Neuroscience, Mount Sinai School of Medicine, New York, New York, United States of America; 2 Laboratory of Mass Spectrometry and Gaseous Ion Chemistry, The Rockefeller University, New York, New York, United States of America; 3 Ben May Department for Cancer Research, The University of Chicago, Chicago, Illinois, United States of America; Boston University School of Medicine, United States of America

## Abstract

**Background:**

Recent studies show that mutations in *L*eucine *R*ich *R*epeat *K*inase 2 (*LRRK2*) are the cause of the most common inherited and some sporadic forms of Parkinson's disease (PD). The molecular mechanism underlying the pathogenic role of LRRK2 mutations in PD remains unknown.

**Methodology/Principal Findings:**

Using affinity purification and mass spectrometric analysis, we investigated phosphorylation sites and binding proteins of LRRK2 purified from mouse brain. We identified multiple phosphorylation sites at N-terminus of LRRK2 including S910, S912, S935 and S973. Focusing on the high stoichiometry S935 phosphorylation site, we developed an anti-pS935 specific antibody and showed that LRRK2 is constitutively phosphorylated at S935 in various tissues (including brain) and at different ages in mice. We find that 14-3-3 proteins (especially isoforms γ and η) bind LRRK2 and this binding depends on phosphorylation of S935. The binding of 14-3-3, with little effect on dimer formation of LRRK2, confers protection of the phosphorylation status of S935. Furthermore, we show that protein kinase A (PKA), but not LRRK2 kinase itself, can cause the phosphorylation of LRRK2 at S935 *in vitro* and in cell culture, suggesting that PKA is a potential upstream kinase that regulates LRRK2 function. Finally, our study indicates that the common PD-related mutations of LRRK2, R1441G, Y1699C and G2019S, decrease homeostatic phosphorylation levels of S935 and impair 14-3-3 binding of LRRK2.

**Conclusions/Significance:**

LRRK2 is extensively phosphorylated *in vivo*, and the phosphorylation of specific sites (e.g. S935) determines 14-3-3 binding of LRRK2. We propose that 14-3-3 is an important regulator of LRRK2-mediated cellular functions. Our study suggests that PKA, a cAMP-dependent kinase involved in regulating dopamine physiology, is a potential upstream kinase that phosphorylates LRRK2 at S935. Furthermore, the reduction of phosphorylation/14-3-3 binding of LRRK2 due to the common familial PD-related mutations provides novel insight into the pathogenic mechanism of LRRK2-linked PD.

## Introduction

Parkinson's disease (PD) is a major human neurodegenerative disease clinically characterized by the motor function deficits as well as non-motor impairment. The most prominent neuropathological features are the loss of midbrain dopaminergic neurons and deposit of intracellular Lewy Body consisting mainly of α-synuclein. The pathogenic mechanism of PD remains largely undefined. However, the recent identification of genetic mutations that are associated with familial PD provides an entry to uncover cellular and molecular pathways that lead to the disease. Mutations in *L*eucine-*R*ich *R*epeat *K*inase 2 (*LRRK2*) have been linked to the most common familial form and some sporadic forms of PD [1], [2]. Because many LRRK2 mutation carriers exhibit typical PD symptoms indistinguishable from idiopathic PD cases, it is hypothesized that an understanding of the biology and pathophysiology of LRRK2 will provide new opportunities to develop effective treatments of PD.

LRRK2 is a large complex protein of 280 kD containing two important enzymatic domains, serine/threonine kinase and ROC GTPase; it also carries several conserved protein motifs including leucine-rich repeats (LRR), the C-terminal of ROC (COR) domain and a WD40 repeat. Previous biochemical analysis demonstrates the kinase and GTPase activity of LRRK2, and these activities are apparently altered by several PD-linked mutations [3], [4], [5], [6], leading to the hypothesis of “gain-of-function” in LRRK2 mutants with enhanced kinase activity [5], [7], [8]. LRRK2 protein can be modified posttranslationally (e.g. by phosphorylation) and forms a dimer *in vitro* and *in vivo* [9], [10], [11]. These alterations may play a critical role in regulating LRRK2 biochemical activities and cellular functions. However, until now little has been known about the normal function of LRRK2 or the pathogenic pathways mediated by PD-linked mutant LRRK2. Several studies show that the mutants of LRRK2 impair the outgrowth or maintenance of neurites in primary neuronal cultures, whereas reduced or abolished expression of LRRK2 has the opposite effect. In addition, inhibition of kinase activity seems to alleviate the toxic phenotype caused by LRRK2 mutants [8], [12], [13], [14].

To elucidate the cellular pathway and pathogenic role of LRRK2 in PD, we investigated LRRK2 protein modifications and interactors in the brain. We show that LRRK2 is phosphorylated at multiple sites. Our study reveals that 14-3-3s bind LRRK2 and the binding depends on the phosphorylation of S935. Furthermore, we show that protein kinase A (PKA) causes phosphorylation of LRRK2 at S935 *in vitro* and in cell culture, implicating PKA pathway in regulating LRRK2 function. Finally, our study suggests that common PD mutations of LRRK2 impair phosphorylation levels of S935 as well as14-3-3 binding. Our data, therefore, provide molecular insight into the regulation of LRRK2 and suggests a potential mechanism for LRRK2-mediated PD pathogenesis.

## Results

### Identification of phosphorylation sites in LRRK2 from mouse brain

**Figure 1:**
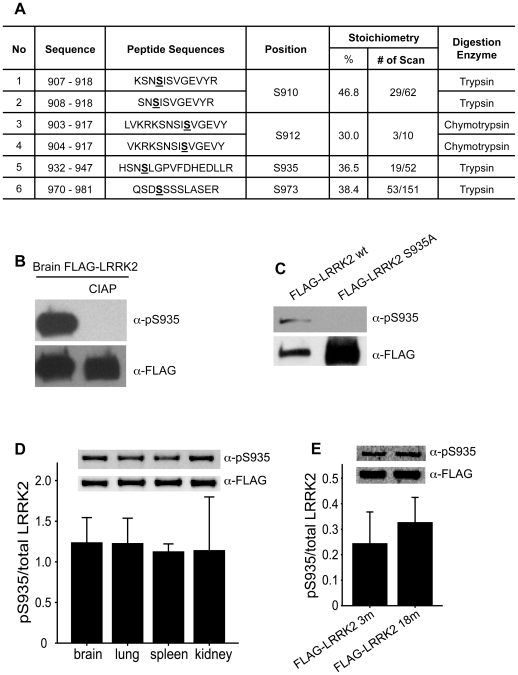
Identification of LRRK2 phosphorylation sites in BAC transgenic brain. (A) Table of the identified phosphorylation sites within tryptic peptides from purified brain FLAG-LRRK2 protein. The estimate of the stoichiometry of phosphorylation is based on the ratio of number of MS/MS spectra observed. The exact location of each proteolytic peptide is shown in the position column. (B) Purified brain FLAG-LRRK2 protein was treated with or without Calf-intestinal alkaline phosphatase (CIAP), followed by Western blot analysis with anti-pS935 antibody or anti-FLAG antibody. (C) FLAG-LRRK2 WT or FLAG-LRRK2 S935A mutant plasmid was transfected into HEK-293T cells; FLAG-tagged proteins were then immunoprecipitated and analyzed by anti-pS935 antibody or anti-FLAG antibody via Western blot analysis. (D) FLAG-LRRK2 was purified from different BAC-transgenic tissues and western blot was performed to detect pS935 and total FLAG-LRRK2 levels. No significant difference in the ratio of pS935/total LRRK2 was observed between different tissues. Data are presented as mean value (± SEM) from three mice. (E) FLAG-LRRK2 was purified from 3 months and 18 months transgenic mouse brain and western blot was performed to detect pS935 and total FLAG-LRRK2 levels. No significant difference in the ratio of pS935/total LRRK2 was observed between 3 months and 18 months. The Western blot signals were quantified by using LI-COR and Odyssey software. Data are presented as mean value (± SEM) from three mice.

We previously reported the purification of FLAG-tagged LRRK2 protein from BAC transgenic mice [3]. For phosphorylation site identification, the purified LRRK2 protein was digested in-gel using various proteases and the resulting proteolytic peptides were analyzed by multiple mass spectrometer methods including MALDI-QqTOF, MALDI-ion trap (LCQ DECA XP), and nano-HPLC/ velos LTQ Orbitrap. The resulting MS/MS data were used to identify proteins and protein modifications. The results reveal 3 serine phosphorylation sites (S910, 935 and 973) from tryptic peptides and 1 serine phosphorylation site (S912) in chymotryptic peptides of LRRK2, respectively (Figure 1A) (Figures S1, S2, S3 and S4). Interestingly, stoichiometry of all 4 serine phosphorylation appears high, as the ratios of MS/MS spectra for modified peptides versus unmodified peptides are all more than 30%. This result indicates the relative high probability of LRRK2 phosphorylation at these sites in the brain (Figures S1, S2, S3 and S4).

In this initial study, we focused on the analysis of the high stoichiometry S935 phosphorylation. We developed an antibody raised against phosphorylated S935 (pS935) peptide. The anti-pS935 antibody detects a strong signal in purified FLAG-LRRK2 protein from BAC transgenic brains, while the signal is completely abolished upon the treatment with calf-intestinal alkaline phosphatase (CIAP) (Figure 1B). The loss of phosphorylation at S935 with alkaline phosphatase treatment was also confirmed by mass spectrometric analysis (Figure S5). The antibody also detected pS935 signal in FLAG-LRRK2 protein isolated from transfected HEK-293T cells. In contrast, FLAG-LRRK2 mutant S935A, where serine 935 was replaced with alanine, was not recognized by this antibody, even though much more mutant protein (compared to wild type) was loaded in the gel (Figure 1C). We also examined the pS935 levels in purified FLAG-LRRK2 from different tissues and at different ages in the brain. The results show that FLAG-LRRK2 is phosphorylated at S935 in lung, spleen and kidney in addition to brain, and the pS935 levels relative to the total FLAG-LRRK2 protein amount are comparable among these tissues (Figure 1D). Moreover, the relative levels of pS935 do not change significantly at different ages in the brain (Figure 1E). The above results suggest that pS935 are maintained at a constant level under normal condition.

### Identification of 14-3-3s in LRRK2 protein complex and specific 14-3-3 isoforms as preferential LRRK2-binding proteins

**Figure 2:**
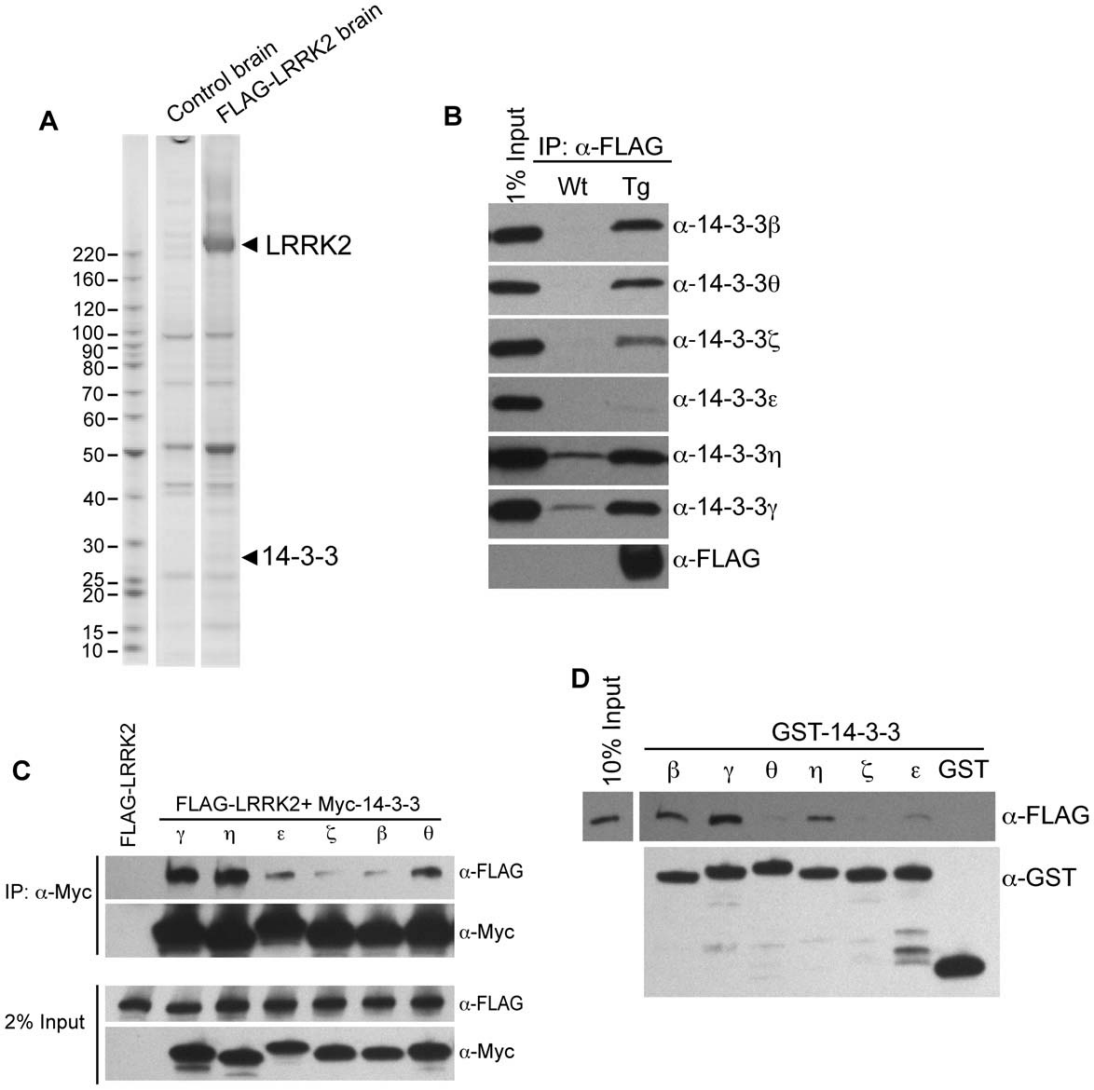
Identification of 14-3-3s as LRRK2 interaction proteins. (A) Coomassie blue staining showing the affinity-purified FLAG-LRRK2 and its binding proteins from FLAG-LRRK2 transgenic brain. The positions of the FLAG-LRRK2 and 14-3-3 bands are labeled with arrows. Wild-type mouse brain was used as the control for background proteins during purification. (B) Western blot analysis was performed to detect the presence of various 14-3-3 isoforms in association with the affinity purified FLAG-LRRK2 protein. Antibodies against specific isoforms of 14-3-3 were used. Wild type mouse tissue was used as the control. (C) FLAG-LRRK2 and different Myc-14-3-3 isoform plasmids were co-transfected into HEK-293T cells and Immunoprecipitation was performed using anti-Myc antibody, followed by the analysis of protein levels using indicated antibodies. (D) FLAG-LRRK2 plasmid was transfected into HEK-293T cells and transfected cell lysate was incubated with different purified GST-14-3-3 isoforms as indicated. The GST-pull down assay was assayed by Western blot analysis of the FLAG-LRRK2.

We sought to identify LRRK2-binding proteins in the brain by analyzing proteins that were affinity-isolated with FLAG-LRRK2 from BAC transgenic brain. We isolated only the proteins unique to the transgenic (compared to non-transgenic control) and performed mass spectrometry analysis. We identified several isoforms of 14-3-3 proteins, such as γ, η, ζ and ε (Figure 2A, arrow) (Figures S6, S7 and S8) that are specifically isolated by FLAG-LRRK2. Using commercial 14-3-3 isoform-specific antibodies, we found 14-3-3γ, η, ζ as well as β, θ in the immunoprecipitated products (Figure 2B). To further evaluate various 14-3-3 isoform binding to LRRK2, we co-expressed FLAG-LRRK2 with individual myc-tagged 14-3-3 isoforms in HEK-293T cells and tested their binding by co-immunoprecipitation (co-IP) analysis. The results indicated that, although all six isoforms co-IP with LRRK2, the γ and η forms pulled down much more LRRK2 protein than did the other isoforms (Figure 2C). In a parallel experiment, we incubated each purified GST-14-3-3 isoform protein with BAC-FLAG-LRRK2 transgenic mouse brain lysate and, followed by a GST pull-down assay. The result again showed enhanced binding of 14-3-3γ and η with LRRK2, compared to 14-3-3ζ, ε and θ (Figure 2D). The above data collectively suggests that LRRK2 prefers 14-3-3γ and η as binding partners and that the binding is likely mediated through direct protein-protein interactions.

### Binding of 14-3-3 to LRRK2 depends on phosphorylation of S935

**Figure 3:**
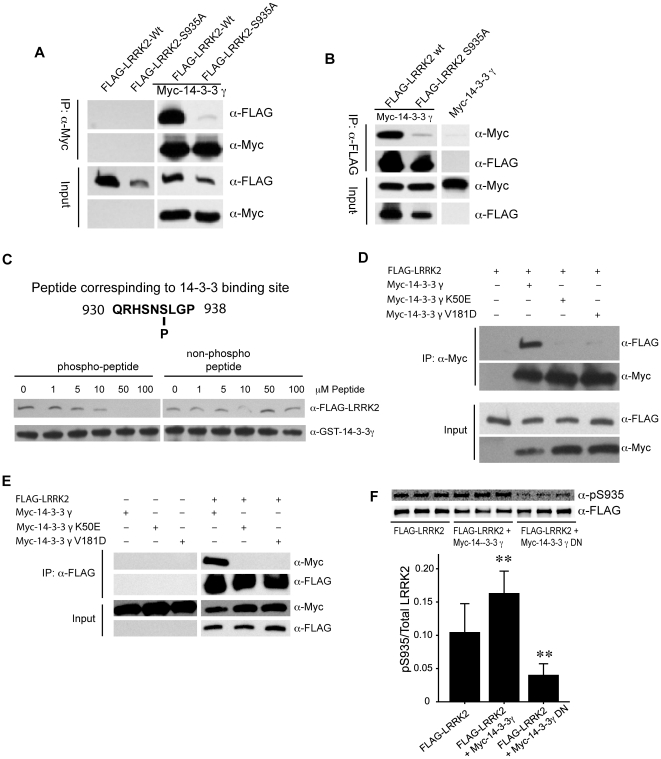
14-3-3 binding to LRRK2 S935 is phosphorylation-dependent. (A, B) FLAG-LRRK2 Wt or S935A mutant plasmid was transfected with or without Myc-14-3-3γ into HEK-293T cells. Co-immunoprecipitation experiments were performed using either anti-myc (A) or anti-FLAG (B) antibody, followed by Western blot analysis using the indicated antibodies. (C) Sepharose bead-conjugated GST-14-3-3γ was incubated with FLAG-LRRK2 transgenic brain lysate in the presence of various concentrations of phosphorylated peptide (pS935) or non-phosphorylated peptide. The binding FLAG-LRRK2 protein was pulled down and analyzed by Western blot analysis using anti-FLAG antibody. (D, E) FLAG-LRRK2 was transfected alone, with Myc-14-3-3γ Wt, with Myc-14-3-3 g K50E mutant, or with Myc-14-3-3γ V181D mutant into HEK-293T cells. The co-IP assay was performed using either anti-Myc (D) or anti-FLAG (E) antibody, followed by Western blot analysis using indicated antibodies. (F) FLAG-LRRK2 was transfected alone, with Myc-14-3-3γ wt or Myc-14-3-3γ dominant negative (DN) mutant (K50E/V181D) into HEK-293T cells. The total FLAG-LRRK2 was immunoprecipitated and analyzed by Western blot using anti-pS935 and anti-FLAG antibodies. The Western blot shows the results from three independent transfections. The quantification of the signals was done by using LI-COR imaging and Odyssey software. The data was analyzed by *One-way ANOVA* (** *P*<0.01). Data are presented as mean value (± SEM) from three independent experiments.

14-3-3 proteins are a family of conserved regulatory molecules that control various cellular functions by directly binding target signaling proteins, including kinases, phosphatases and receptors [15]. 14-3-3 binding was found frequently in a phospho-serine dependent manner and occurs in a consensus motif (RSXpSXP and RXY/FXpSXP) [16]. We next investigated whether pS935 of LRRK2 is relevant to the binding of 14-3-3 (although S935 surrounding sequence does not fit well with the known consensus motif). First, we transfected HEK-293T cells with plasmids containing Myc-14-3-3γ and FLAG-LRRK2 or FLAG-LRRK2-S935A. We found that anti-Myc antibody co-immunoprecipitated 14-3-3γ with FLAG-LRRK2 but not FLAG-LRRK2-S935A (Figure 3A). Reciprocal Co-IP assay using anti-FLAG antibody showed a similar result that 14-3-3 was pulled down only with LRRK2 wild type but not S935A mutant (Figure 3B). To further test the role of phophorylated S935 in binding, we incubated purified GST-14-3-3γ and BAC-FLAG-LRRK2 transgenic mouse brain lysate in the presence of phospho-peptide QRHSNpSLGP (containing pS935) and non-phospho-peptide QRHSNSLGP (control) at various concentrations. GST-14-3-3γ pulled down FLAG-LRRK2 in the absence of the peptides; however, adding the pS935-containing peptide effectively inhibited the co-precipitation of GST-14-3-3γ with FLAG-LRRK2 in a dosage-dependent manner. By contrast, the control peptide has no inhibitory effect toward the pull-down of GST-14-3-3γ and FLAG-LRRK2 under the same conditions (Figure 3C). These results indicate that the phospho-peptide (but not the control peptide) competed for 14-3-3 binding, therefore demonstrating the critical role of pS935 in 14-3-3 binding with LRRK2.

Furthermore, we co-transfected FLAG-LRRK2 with Myc-14-3-3γ wild type, mutant K50E or V181D, which was reportedly impaired in binding phospho-serine of target proteins [17], [18]. In contrast to wild type 14-3-3, mutant K50E or V181D completely lost the binding to LRRK2 as shown in experiments that either use anti-FLAG or anti-Myc antibody for the co-IP (Figure 3 D, E), consistent with the notion that 14-3-3 binding depends on phospho-serine in LRRK2.

To study the relationship of 14-3-3 binding and S935 phosphorylation, we transfected FLAG-LRRK2 in the absence or presence of 14-3-3 wild type or mutant K50E/V181D, which was previously shown as a dominant negative mutant (DN) that abolishes wild type14-3-3 binding to the target proteins [19]. The result showed that the relative pS935 levels, as measured with anti-pS935 staining over anti-FLAG staining, were significantly higher with co-transfection of Myc-14-3-3γ and FLAG-LRRK2 than FLAG-LRRK2 transfection alone. In contrast, co-transfection of FLAG-LRRK2 with Myc-14-3-3 DN markedly reduced the relative pS935 levels of LRRK2 (Figure 3F). These results suggest that 14-3-3 binding prevents the dephosphorylation of LRRK2 at S935.

### S935A mutation does not affect LRRK2 dimerization

**Figure 4:**
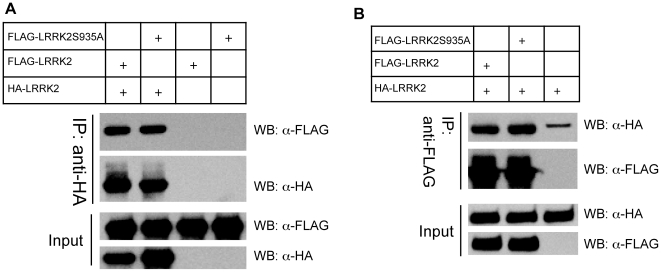
S935A mutation does not affect LRRK2 dimerization. (A, B) FLAG-LRRK2 WT or S935A mutant construct was co-transfected with HA-LRRK2 constructs. The Co-IP assay was performed using either anti-HA (A) or anti-FLAG (B) antibody, followed by Western blot analysis using the indicated antibodies.

One of the important roles of 14-3-3 binding is to provide structural support for the target protein dimer formation via the self-dimerization of 14-3-3 proteins [20]. Since LRRK2 is known to form dimers *in vitro* and *in vivo* [9], [10], [11], we next investigated whether 14-3-3 interaction of LRRK2 is important for the dimerization of LRRK2. HEK-293T cells were co-transfected with HA-tagged LRRK2 and FLAG-LRRK2 or FLAG-LRRK2 S935A mutant. The reciprocal co-IP experiments show that FLAG-LRRK2 wild type and FLAG-LRRK2 S935A mutant did not differ in pulling down together with HA-LRRK2 (Figure 4A, B). Considering S935A mutant of LRRK2 is defective in phosphorylation at 935 and 14-3-3 binding, this result suggest that 14-3-3 binding to LRRK2 does not play a critical role in LRRK2 dimer formation. Rather, the dimerization of LRRK2 is perhaps determined by the sequence outside 14-3-3 binding region [9], [10], [11].

### Common familial PD mutations of LRRK2 cause a reduction in pS935 levels and 14-3-3 binding

**Figure 5:**
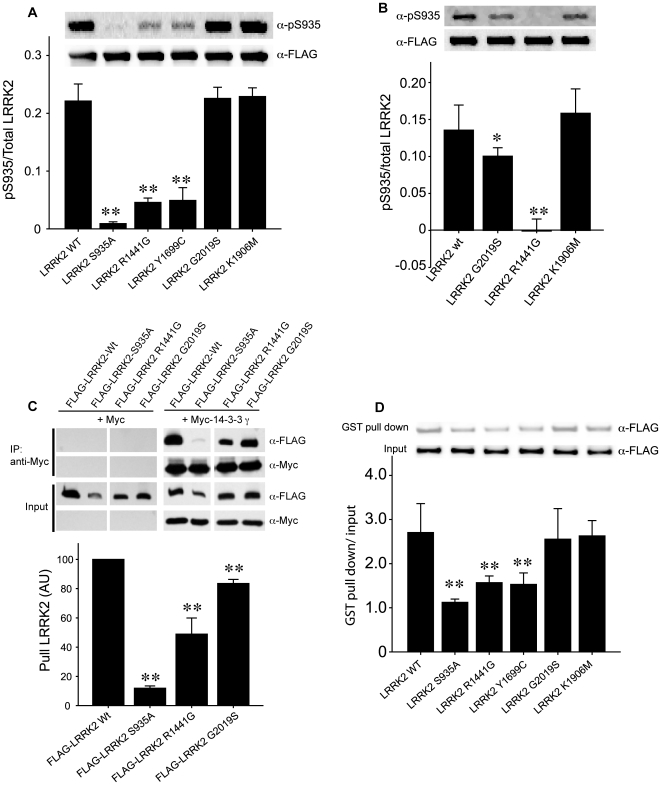
PD-linked mutants affect S935 phosphorylation and 14-3-3 binding of LRRK2. (A) FLAG-LRRK2 WT, S935A, R1441G, Y1699C, G2019S or K1906M mutant plasmid was transfected into HEK-293T cells. The LRRK2 variants were immunoprecipitated and analyzed by Western blot using anti-pS935 and anti-FLAG antibodies. Quantification of the signals was performed using LI-COR and Odyssey software and the data was analyzed by *One-way ANOVA* (** *P*<0.01). Data are presented as mean value (± SEM) from three independent experiments. (B) FLAG-LRRK2-WT, G2019S, R1441G and K1906M was immunoprecipitated from the brain lysates of the corresponding BAC-transgenic mice. The quantification of the signals was performed as described above. The ratio of pS935 signal over total LRRK2 signal is shown. The data was analyzed with *One-way ANOVA* (* *P*<0.05, ** *P*<0.01). Data are presented as mean value (± SEM) from three mice. (C) Myc-14-3-3γ (or control Myc vector) was co-transfected with FLAG-LRRK2 Wt, S935A, R1441G or G2019S mutant plasmid into HEK-293T cells. Co-IP was performed by using anti-Myc antibody. The pulled down LRRK2 variant levels were analyzed by Western blot analysis with the indicated antibodies and quantified using LI-COR and Odyssey system. The data was analyzed by *One-way ANOVA* (** *P*<0.01). Data are presented as mean value (± SEM) from three independent experiments. (D) FLAG-LRRK2-WT, S935A, R1441G, Y1699C, G2019S or K1906M mutant plasmid was transfected individually into HEK-293T cells. Sepharose 4B beads-conjugated GST-14-3-3γ protein was incubated with different transfected cell lysates containing LRRK2-Wt or various mutants. Western blot analysis was performed to determine the LRRK2 variant amount after the GST pull-down assay. The results were quantified as described above. The ratio of pulled down LRRK2 and input LRRK2 was measured and analyzed by *One-Way ANOVA* (** *P*<0.01). Data are presented as mean value (± SEM) from three independent experiments.

Binding of 14-3-3 with various kinases plays an important role in regulating downstream signaling [15], [19]. Although the LRRK2-mediated signaling pathway has not been clearly elucidated, we sought to investigate whether common PD mutations of LRRK2, including G2019S, R1441G and Y1699C, affect S935 phosphorylation and/or 14-3-3 binding of LRRK2. First, we examined the pS935 levels in the PD-linked mutants of LRRK2 expressed in HEK-293T cells. Compared to wild type LRRK2, mutants R1441G and Y1699C isolated from cells have markedly reduced pS935 levels; mutant G2019S and kinase-deficient mutant K1906M did not change significantly in pS935 levels (Figure 5A). However, when we assayed pS935 levels in LRRK2 wild type or mutant proteins isolated from transgenic brains [21], G2019S mutant showed a modest but significant decrease in pS935 levels as compared to LRRK2 wild type (Figure 5B). R1441G mutant from transgenic brain again exhibited a marked reduction of pS935 levels, whereas K1906M had little change in pS935 levels in the brain (Figure 5B). Next, we examined 14-3-3 binding with LRRK2 variants in transfected cells. We transfected HEK-293T cells with Myc-14-3-3γ and FLAG-LRRK2 wild type or various mutant. Compared to wild type LRRK2, all three mutants (S935A, R1441G and G2019S) exhibited reduced co-immunoprecipitation with Myc-14-3-3γ, suggesting that these mutants are impaired in 14-3-3 binding. But the degree of impairment in these mutants differs, with S935A affected the most (∼90% reduction) and G2019S the least (<20%) (Figure 5C).

We further evaluated the alteration of PD mutants in 14-3-3 binding *in vitro*. We incubated purified GST-14-3-3γ proteins with extracts from HEK-293T cells transfected with FLAG-LRRK2 wild type or various mutants, followed by analysis of FLAG-LRRK2 protein levels pulled down by GST-14-3-3γ. The results showed that binding of 14-3-3 to mutant R1441G or Y1699C is markedly reduced as compared to wild type LRRK2, whereas 14-3-3 binding is affected little in G2019S or K1906M mutant (Figure 5D). The above results indicate a general effect of the above PD-linked mutations on the phosphorylation of S935 and 14-3-3 binding: the reduction of pS935 levels and 14-3-3 binding caused by R1441G and Y1699C is consistent in various assays; while the inhibitory effect of G2019S is modest and assay-dependent. The disparity in G2019S results may reflect the different sensitivity of the various assay systems. It is noteworthy that the K1906M mutation did not alter the levels of pS935 or 14-3-3 binding of LRRK2 in all assays, suggesting that the LRRK2 kinase activity is not primarily responsible for the phosphorylation of S935 (Figure 5A, B, D).

### Protein kinase A (but not LRRK2 kinase) causes phosphorylation of S935 *in vitro* and in cell culture

**Figure 6:**
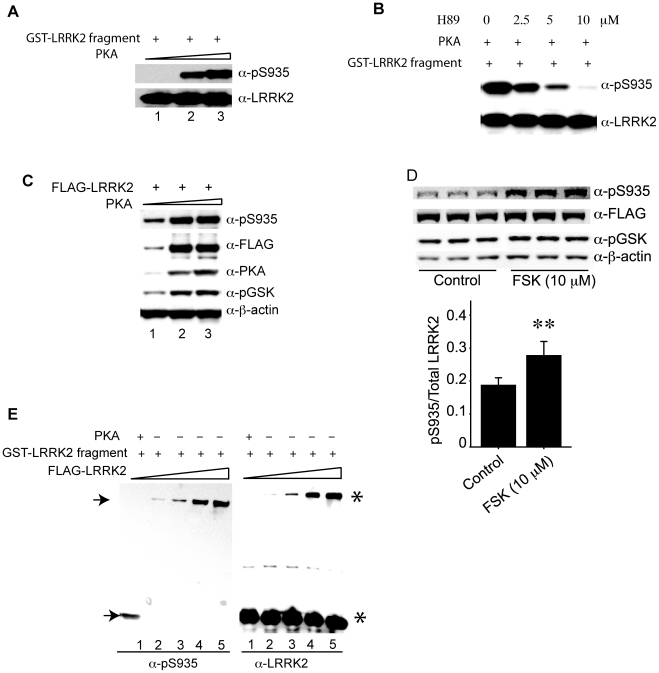
PKA, not LRRK2 itself, can phosphorylate S935 in vitro and within cells. (A) 0.4 µg of purified GST-LRRK2 fragments (800–1000aa) was incubated with different amount of the PKA catalytic subunit (lane 1: without PKA; lane 2: 2500 units PKA; lane 3: 10,000 units PKA). The reaction was stopped by adding 3× SDS-PAGE sample buffer and Western blot was performed by using anti-pS935 and anti-LRRK2 antibodies. (B) Different concentrations of H-89 (0, 2.5, 5 and 10 µM) were added to the PKA and GST-LRRK2 fragment reaction mixture, and Western blot analyses was performed by using anti-pS935 and anti-LRRK2 antibodies. (C) FLAG-LRRK2 was co-transfected with different amounts of PKA plasmid into HEK-293T cells (The ratio of FLAG-LRRK2 verse PKA plasmid DNA are: 0 in lane 1, 1∶1 in lane 2 and 1∶10 in lane 3). The transfected cell lysate was harvested and analyzed by Western blot using anti-pS935, anti-FLAG, anti-PKA, anti-pGSK and anti-β-actin antibodies. (D) FLAG-LRRK2 was transfected into HEK-293T cells and after 40 hours transfection, 10 µM FSK was added to the cell culture medium and incubated for 30 min. Then the cells were harvested and western blot analysis was performed to study various protein levels by using anti-pS935, anti-FLAG, anti-pGSK and anti-β-actin antibodies. The pS935 LRRK2 and total LRRK2 signals were quantified by using the LI-COR Odyssey software system and the ratio of pS935 and total LRRK2 was calculated and analyzed by *One-way ANOVA* (** *P*<0.01). Western blot results of three independent experiments are shown, and data are presented as mean value (± SEM) from three independent experiments. (E) GST-LRRK2 fragment (800–1000aa) protein was incubated with purified FLAG-LRRK2 (from mouse brains) or with PKA catalytic enzyme subunit (as positive control) (lane 1: 2500 units PKA; lane 2: 12.5 nM FLAG-LRRK2; lane 3: 25 nM FLAG-LRRK2; lane 4: 125 nM FLAG-LRRK2; lane 5: 250 nM FLAG-LRRK2) in Kinase assay buffer and western blot was performed by using anti-pS935 and anti-LRRK2 antibodies. The arrows indicated the phosphorylated form of full length LRRK2 and GST-LRRK2 fragment; the asterisks indicated the total full length LRRK2 and total GST-LRRK2 fragment, respectively.

As LRRK2 kinase activity is unlikely the cause for the phosphorylation of S935 on its own (Figure 5), we sought to identify other possible kinases that may be responsible for phosphorylation of S935. A previous study showed that cAMP-dependent protein kinase (PKA) could phosphorylate LRRK2 *in vitro* but without any knowledge about the phosphorylation sites [22]. We therefore performed kinase assay with purified PKA catalytic subunit and GST fusion protein containing LRRK2 fragment (800–1000aa). The result revealed that PKA phosphorylates S935 as detected with anti-pS935 antibody and that the phosphorylation is enhanced with increased input of PKA amount (Figure 6A). In addition, the phosphorylation of S935 is inhibited by PKA inhibitor H89 [23] (Figure 6B). Next, we tested whether or not PKA mediates phosphorylation of S935 in cells. Co-expression of FLAG-LRRK2 and PKA catalytic domain resulted in a significant increase in pS935 levels as compared to that with the transfection of FLAG-LRRK2 alone. Again, the phosphorylation levels are enhanced with increasing transfected PKA plasmid amount (Figure 6C). Phosphorylation of GSK, a known substrate of PKA, is used for the positive control of PKA activity in this assay. Furthermore, we used PKA activator forskolin (FSK) to treat cells expressing FLAG-LRRK2. The result showed that administration of FSK at 10 µM caused a significant increase in pS935 levels of LRRK2 likely as a result of enhanced PKA activity as indicated by the increase in pGSK levels (Figure 6D). Finally, in an *in vitro* kinase assay as described above (Figure 6A), we found that, while purified PKA directly phosphorylates S935 in GST-LRRK2 fragment, purified FLAG-LRRK2 kinase does not cause any change in pS935 levels in GST-LRRK2 fragment (Figure 6E), despite the active kinase activity of FLAG-LRRK2 towards generic substrate MBP (data not shown) [3]. This result further demonstrates that LRRK2 kinase is not responsible for pS935, whereas PKA is a potential upstream kinase that can cause the phosphorylation of LRRK2 at S935.

## Discussion

Our study identifies serial molecular events related to the regulation of LRRK2: multiple novel phosphorylation sites (S910, S912, S935 and S973, all in the N-terminal region), potential phosphorylation of S935 by PKA, and pS935-dependent 14-3-3 binding of LRRK2. These events may represent an important part of a signaling pathway in regulating LRRK2 cellular function under physiological conditions. Furthermore, we show that the phosphorylation status of S935 and 14-3-3 binding of LRRK2 is impaired by the most common familial mutations including G2019S, R1441G and Y1699C. This result provides a new avenue for the study of pathogenic mutations of LRRK2 in PD.

Interestingly, while our study was in final stage of submission, Nichols and Alessi's group reported the phosphorylation of LRRK2 at S910, S935 and S973, and pS910/pS935-dependent 14-3-3 binding of LRRK2. Their study indicated a nearly identical role of S910 and S935 in 14-3-3 binding [24], [25]. Furthermore, they examined 41 known LRRK2 mutations and found that five out of six pathogenic mutations (except G2019S) have markedly reduced phosphorylation of S910/S935 and thereby disrupted 14-3-3 binding of LRRK2. Therefore, our study and theirs independently demonstrate the phosphorylation-dependent binding of 14-3-3 to LRRK2, which may play a critical role in regulating LRRK2 function *in vivo*; PD-linked mutations impair 14-3-3 interaction with LRRK2 and thus disturb the regulation of LRRK2 by 14-3-3.

**Figure 7:**
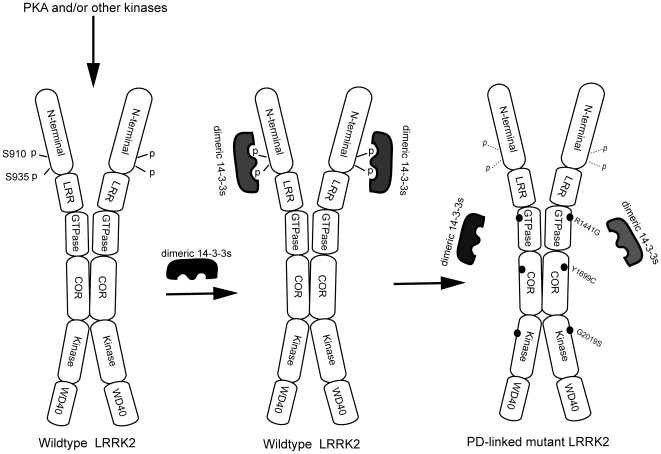
PKA Phosphorylation, 14-3-3 binding of LRRK2, and the effect of common familial mutations of LRRK2 in 14-3-3 binding. A schematic model showing PKA (or other kinase) phosphorylation of S910/S935, dimeric14-3-3 binding of LRRK2 at pS910/pS935 sites in wild type LRRK2; PD-linked mutations R1441G, Y1699C or G2019S abolishes or reduces phosphorylation of S910/S935 and impairs 14-3-3 binding. In addition, we propose that dimeric 14-3-3 bind to pS910 and pS935 in the same LRRK2 molecule and binding of 14-3-3 plays little role in LRRK2 dimer formation.

Our study also provides additional insight into the functional relevance of the LRRK2-14-3-3 interaction. In particular, our results indicate that the most common mutation G2019S also causes a reduction of pS935 levels in LRRK2 from transgenic brain, despite little effect of G2019S seen in LRRK2 protein isolated from cell culture. Our study has further characterized the specific isoforms of 14-3-3 binding of LRRK2 and shown the relevance of 14-3-3 binding to the dimer formation of LRRK2. Thus, there are seven isoforms of 14-3-3 proteins and many 14-3-3 target proteins only bind to selective isoforms of 14-3-3 [26], [27], [28]. Although several isoforms 14-3-3 were identified through our mass spectrometric analysis, our data suggest that LRRK2 binds preferentially to γ and η form (both are abundant in the brain). This information is important for future biochemical and structural analyses of LRRK2-14-3-3 interactions. We have also sought to investigate whether 14-3-3 is a potential substrate of LRRK2, because previous studies had shown that 14-3-3 is a phosphoprotein [29], [30]. Our data indicated that purified LRRK2 efficiently phosphorylates generic substrate MBP but not 14-3-3 (data not shown). In addition, because a body of evidence has indicated an important role of 14-3-3 in assisting target protein dimerization [19], we tested whether 14-3-3 regulates LRRK2 dimer formation. Our data reveal that the LRRK2 mutant defective in phosphorylation of S935/14-3-3 binding is not affected in dimer formation. The result suggests that 14-3-3 does not play a critical role in dimerization of LRRK2; rather, dimerization of LRRK2 is mediated primarily by the sequence outside of the S910/S935 sites [9], [10], [11]. This result, along with the previous report, is consistent with a model that homo or hetero-dimeric 14-3-3 bind to two separate, neighboring phosphorylation sites (e.g. pS910 and S935) on the same LRRK2 molecule, and disruption of either phosphorylation site would destabilize dimeric 14-3-3 binding of LRRK2 (Figure 7) [25].

We have also examined the potential role of 14-3-3 binding in directing LRRK2 subcellular localization. However, overexpression of wild type LRRK2 or S935A mutant results in largely diffuse localization of each protein in COS-7 or HeLa cells, thus no obvious difference was observed between the wild type and S935A mutant of LRRK2 (our unpublished results). This result, however, is in contrast to the recent report that S935A mutant was associated with punta localization in COS-7 cells [24], [25]. It is unclear what causes the discrepancy of the subcellular localization of LRRK2 mutants in the two studies, despite that 14-3-3 binding of S935A mutant LRRK2 was impaired in both studies. An important question that remains to be answered is whether 14-3-3 binding represents a mechanism for LRRK2 trafficking or distribution at different subcompartments of neurons.

Finally, our study reveals a potential link of PKA kinase to the phophorylation of LRRK2 at S935, therefore adding a new member to the list of known substrates of PKA that are also 14-3-3 targets [31], [32], [33], [34]. One such an example is the PKA-mediated phosphorylation of S413 and 14-3-3 binding of pS413 in RIM1alpha, which is an active zone protein important for synaptic transmission [35], [36]. Future study should investigate in detail whether PKA as well as other kinase activities are responsible for phosphorylation of S910, S912, S935 or S973. Furthermore, previous evidence indicated that the effect of dopamine is largely mediated through cAMP/PKA signaling cascade [37], [38]. Given the observation of dopamine transmission deficits in several LRRK2 mutant rodent models [21], [39], [40], [41], the potential connection of PKA-LRRK2 signaling indicates an attractive cellular pathway for the future dissection of the molecular mechanism underlying LRRK2 mutations in the pathogenesis of PD.

## Materials and Methods

### Reagents

Modified trypsin and EDTA-free protease inhibitor cocktail tablets were purchased from Roche Diagnostics (Indianapolis, IN). Modified porcine trypsin and chymotrypsin were purchased from Promega Inc. (Madison, WI). LC/MS grade water and acetonitrile (ACN) were purchased from Honeywell Burdick & Jackson (Muekegon, MI). Protein inhibitor tablets and CIAP were purchased from Roche (Branchbury, NJ); FLAG-M2 beads, H89 and Forskolin (FSK) were purchased from Sigma (St. Louis, MO); Site-mutagenesis kit was purchased from Stratagene (Santa Clara, CA); NuPAGE Bis-Tris gels, MOP SDS running buffer, antioxidant, and lipofectamine 2000 kit were purchased from Invitrogen (Carlsbad, CA). GelCode Blue Stain Reagent, Trifluoroacetic acid and Tris (2-carboxyethyl)-phosphine hydrochloride were purchased from Pierce (Rockford, IL). The following antibodies were used in this study: FLAG (Sigma, St. Louis, MO), Myc, pGSK-3-alph/beta (S21/9), β-actin and PKA C-alpha (Cell Signaling, Danvers, MA), 14-3-3 isoforms and HA (Santa Cruz, Ca), Phospho-S935 (developed with Cocalico Biological Inc. Reamstown, PA). PKA catalytic subunit was purchased from New England Biolabs (Ipswich, MA). Non-phospho- and Phospho-peptides (QRHSNSLGPC and QRHSNpSLGPC) were synthesized with acetylated N-termini and HPLC purified in The Rockefeller University Proteomics Resource Center.

### Transgenic mice and plasmids

Mice were housed in the Center for Comparative Medicine at Mount Sinai School of Medicine. Handling procedures were in accordance with NIH guidelines and approved by the Institutional Animal Care and Use Committees of the institute (IACUC). The institution has Animal Welfare Assurance on file with the Office for laboratory Animal Welfare. The Assurance number is A3111-01.

BAC transgenic mice expressing FLAG-LRRK2-Wt and FLAG-LRRK2-G2019S were described previously [3], [21]. The BAC mice expressing FLAG-LRRK2-R1441G or FLAG-LRRK2-K1906M were generated using the similar BAC modification strategy. The following plasmid vectors were used to express LRRK2, 14-3-3 isoforms and LRRK2 fragment (800–1000aa): 3×FLAG and 3×Myc plasmid were purchased from Sigma (St. Louis, MO). HA vector was purchased from Santa Cruz (Santa Clara, CA). GST vector was purchased from (GE Healthcare, Piscataway, NJ). Full-length LRRK2 cloning method was described in previous report. The mutations (S935A, R1441G, Y1699C, K1906M and G2019S) were generated by using the Quick-change site-directed mutagenesis kit.

### Affinity purification of transgenic mouse brain samples

Affinity purification of FLAG-LRRK2 and its interacting proteins was carried out as described in [42], with slight modification. In brief, brain extracts were obtained from non-transgenic and FLAG-LRRK2 mice (P30) by homogenizing 2 brains with a motor-driven homogenizer (speed 2.5, 12 strokes) in 3 ml buffer containing 0.32 M sucrose, 1 mM NaHCO_3_, 20 mM HEPEs/pH 7.4, 1 mM MgCl_2_, 0.25 mM CaCl_2_, EDTA-free protease inhibitor cocktail, 200 µg/mL PMSF, pepstatin 4 µg/mL and DNase I. The tissue extracts were centrifuge at 1,500 g for 5 min and the pellets were homogenized again in 1 ml buffer for 8 strokes and centrifuged again. Supernatants from the two homogenization steps were pooled, centrifuged at 750 g for 10 min twice. The collected supernatants were diluted with equal volumes of 2× pull-out buffer (1× pull-out buffer containing 20 mM HEPEs/pH 7.4, 1 mM MgCl_2_, EDTA-free protease inhibitor cocktail, 200 µg/mL PMSF, 4 µg/mL pepstatin, 0.1% triton X-100 and 150 mM NaCl). Sample equivalent to half brain was incubated with FALG M2 beads (∼10 µl bed volume) for 2 hours at 4°C. After washed 5 times with 1× pullout buffer, beads were eluted with 250 µl elution buffer (0.5 mM EDTA and 0.5 M NH3·H_2_O) at room temperature.

### In-gel digestion of purified LRRK2 protein with trypsin and chymotrypsin

The purified LRRK2 was resolved in SDS-PAGE and visualized by colloidal Coomassie blue staining. The protein band of interest were excised, in-gel digested after reduction and alkylation, and extracted using the clean protocol previously described [43].

### Nano-HPLC/mass spectrometric analysis

The proteolytic peptides derived from LRRK2 were analyzed in Velos LTQ Orbitrap mass spectrometer (Thermo Fisher Scientific, Waltham, MA). The peptides were separated in a home-made capillary HPLC column (110 mm length×75 mm internal diameter, 5 mm particle size, 100 Å pore diameter) with Jupiter C_12_ resin (Phenomenex, St. Torrance, CA) using a gradient from 2% to 30% solvent B in solvent A (mobile phase A: 0% ACN in 0.1% formic acid; and mobile phase B: 100% ACN in 0.1% formic acid) for 50 min. The eluted peptides were directly electrosprayed into the mass spectrometer using a nanospray source. The spray voltage was set to 2.1 kV and the temperature of the heated capillary was set to 300°C. The MS was operated in the data-dependent mode to automatically switch between Orbitrap-MS and Velos LTQ-MS/MS acquisition. Survey full scan MS spectra (from m/z 350–1500) were acquired in the Orbitrap with resolution *R* = 60,000 at *m/z* = 400. The 20 most intense ions were sequentially isolated for fragmentation in the linear ion trap using collision-induced dissociation (normalized collision energy = 35%, activation Q = 0.250 and activation time = 10 ms) in the Velos LTQ. Maximal filling times were 30 ms for the full scans and 25 ms for the MS/MS scans. Precursor ion charge state screening was enabled, and singly and unassigned charge states were rejected. A lock-mass ion from ambient air (m/z 445.120024) was used for internal calibration of all full scan measurements with the Orbitrap detector [43], [44]. The following parameters were specified in MS/MS analysis: dynamic exclusion (36 seconds); the repeat count (2) and the exclusion window (+3 and −1.5 Da).

### Protein sequencing alignment

Mass spectra collected by MALDI-QqTOF and MALDI-ion trap mass spectrometers were analyzed by the computer search engines ProFound (http://prowl.rockefeller.edu/prowl-cgi/profound.exe), Xproteo (http://www.xproteo.com) and GPM (http://prowl.rockefeller.edu/tandem/thegpm_tandem.html) using the NCBI non-redundant mouse protein database. All MS/MS spectra were searched against the IPI-mouse data base (v3.74) protein sequence database (56860 sequences) using Mascot (v2.1). The specific parameters for protein sequence database searching included serine, threonine and tyrosine phosphorylation; cysteine carbamidomethylation and methionine oxidation as variable modifications. Other parameters used in data analysis were: four allowed missing cleavages; mass error of 10 ppm for precursor ions and 0.5 Da for fragment ions. Charge states of +2, and +3 were considered for parent ions. If more than one spectrum was assigned to a peptide, only the spectrum with the highest Mascot score was selected for manual analysis. All peptides identified with peptide scores of mascot >20 were manually examined using rules described previously [45].

### Immunoprecipitation

Immunoprecipitation was described as previously with modification [3]. LRRK2 transgenic brain was homogenized with homogenization buffer (50 mM Tris HCl at pH 7.5, 5% Glycerol, 1 mM NaHCO3, 0.25 mM CaCl_2_, 1 mM MgCl_2_, 1 mM PMSF, 10 µg/mL pepstatin, 20 mM beta-Glycerol phosphate, 1 mM Na Vanadate, 50 mM NaF, 10 mM Pyrophosphate, 0.01%Triton X-100 and mini complete protease inhibitor cocktail), and incubated at 4°C on rotator for 30 min. Transfected HEK-293T cells were harvested and suspended in cell lyses buffer (50 mM Tris HCl at pH 7.5, 150 mM NaCl, 1 mM PMSF, 10 µg/mL pepstatin, 20 mM beta-Glycerol phosphate, 1 mM Na Vanadate, 50 mM NaF, 10 mM Pyrophosphate, 1%Triton X-100 and mini complete protease inhibitor cocktail). The cell lysate or homogenized brain lysate were clarified at 12,000 g for 10 min at 4°C, the 3×FLAG-LRRK2 protein was purified using anti-FLAG Affinity Gel according to the manual. For Myc-14-3-3 immunoprecipitation, the HEK-293T cell lysate was precleared using agarose gel for 1 hour at 4°C, and then anti-Myc antibody was added and incubated for overnight. The beads were washed with modified RIPA buffer (50 mM Tris HCl at pH 7.5, 300 mM NaCl, 10 mM MgCl_2_, 1 mM PMSF, 10 mg/mL pepstatin, 0.5% Sodium Deoxycholate, 0.1% Sodium Dodecyl Sulfate). The immunoprecipitated protein was eluted by using 1× sample buffer.

### GST-pull down assay

GST-14-3-3 isoform protein was expressed in bacteria with 100 µM IPTG induction for 4 hours at 37°C, and purified following the manufacture's instruction (GE Healthcare). The protein concentration was measured on SDS-PAGE and Coomassie Staining comparing to Standard BSA. 5 µg of each GST-14-3-3 isoform protein was incubated with 100 µg FLAG-LRRK2 transfected HEK-293T cell lyses supernatant for 4 hours at 4°C and washed with cell lyses buffer. The bound protein was eluted with 1× SDS-PAGE sample buffer. For the phosphopeptide competition assay, 2 mg of brain lysate from FLAG-LRRK2 Wt transgenic mice was incubated with different concentration of non-phosphopeptide or phophopeptide (S935) for 1 hour. Then GST-14-3-3γ beads were added and incubated for 4 hours. The beads were then washed and the bound protein was eluted with 1× sample buffer.

### PKA kinase assay

GST-LRRK2 fragment (800–1000aa) was expressed in bacteria with 50 µM IPTG induction at RT for overnight and purified following the manufacture's instruction. The protein concentration was measured on SDS-PAGE and Coomassie Staining and comparing to standard BSA. The kinase assay was performed following the New England Biolab protocol. Briefly, 400 ng GST-LRRK2 fragment protein was incubated with different concentration of PKA catalytic subunit protein at 30°C for 20 min. Or GST-LRRK2 fragment protein was incubated with PKA catalytic subunit protein and different concentration of H89 at 30°C for 20 min. The reaction was stopped by adding 6× SDS-PAGE sample buffer, followed by boiling the samples for 5 min.

## Supporting Information

Figure S1 **Identification of LRRK2 S935 phosphorylation by MALDI QqTOF and ion trap mass spectrometers.** MALDI QqTOF mass spectrum of tryptic digested peptides extracted from the affinity purified mouse brain LRRK2 (band shown in Figure 5A). Two tryptic peptides containing the S935 phosphorylation site are labeled A* and B*. The corresponding unphosphorylated peptides are labeled A and B.
(TIF)

Figure S2 **Identification of LRRK2 S935 phosphorylation by MALDI QqTOF and ion trap mass spectrometers.** (A) MALDI ion trap MS/MS spectrum of the phosphorylated peptide HSNpSLGPVFDHEDLLR. (B) MALDI ion trap MS/MS spectrum of the phosphorylated peptide HSNpSLGPVFDHEDLLRR. Both MS/MS spectra show dominant peaks due to characteristic loss of H3PO4 (98 Da). For both (A) and (B), insets show zoomed-in regions of the spectra. Phosphorylated-S935-containing fragment ions are labeled in red.
(TIF)

Figure S3 **Analysis of phosphorylation sites in LRRK2 by nano-HPLC/velos LTQ Orbitrap mass spectrometer.** MS/MS spectra of phosphorylated peptides at serine 910 (SNpSISVGEVYR) (**A**), serine 912 (LVKRKSNSIpSVGEVY) (**B**) in purified LRRK2 protein, respectively. In this study, we digested the purified LRRK2 protein in-gel using trypsin or chymotrypsin. The MS/MS data were analyzed by the Mascot algorithm to identify the protein and its posttranslational modifications. The candidate peptides bearing the serine phosphorylation were further examined manually as previously described [45]. The phosphorylated peptide can be identified by a mass shift of 79.96633 Da at serine/threonine/tyrosine residues.
(TIF)

Figure S4 **Analysis of phosphorylation sites in LRRK2 by nano-HPLC/velos LTQ Orbitrap mass spectrometer.** MS/MS spectra of phosphorylated peptides at serine 935 (HSNpSLGPVFDHEDLLR) (**A**), and serine 973 (QSDpSSSSLASER) (**B**) in purified LRRK2 protein, respectively. In this study, we digested the purified LRRK2 protein in-gel using trypsin or chymotrypsin. The MS/MS data were analyzed by the Mascot algorithm to identify the protein and its posttranslational modifications. The candidate peptides bearing the serine phosphorylation were further examined manually as previously described [45]. The phosphorylated peptide can be identified by a mass shift of 79.96633 Da at serine/threonine/tyrosine residues.
(TIF)

Figure S5 **Confirmation of LRRK2 S935 phosphorylation by a combination of alkaline phosphatase treatment and mass spectrometry.** (**A**) Coomassie blue stain gel showing affinity purified FLAG-LRRK2 and its interacting proteins from FLAG-LRRK2 BAC transgenic mouse brain and lung, with and without alkaline phosphatase (AP) treatment. LRRK2 and AP bands are labeled by arrows. AP treatment was performed by incubating anti-FLAG immunoprecipitation eluent in AP (Roche, 2 U/µL) at 37°C for 1 h. (**B**) MALDI QqTOF mass spectra of LRRK2 tryptic digested peptides extracted from the gel bands shown in (**A**). The monoisotopic peaks of the unphosphorylated and phosphorylated LRRK2 tryptic peptide 932[HSNSLGPVFDHEDLLR]947 are highlighted in yellow. (**C**) MS^3^ tandem mass spectrum confirming phosphorylated LRRK2 tryptic peptide 932[HSNpSLGPVFDHEDLLR]947, using a MALDI ion trap mass spectrometer.
(TIF)

Figure S6 **Identification of 14-3-3 isoforms by MALDI QqTOF and ion trap mass spectrometry.** (A) MALDI QqTOF mass spectrum of tryptic digested peptides extracted from the affinity purified mouse brain 14-3-3 (band shown in Figure 5A). (B) MALDI ion trap MS/MS spectra of unique tryptic peptides of 14-3-3γ.
(TIF)

Figure S7 **Identification of 14-3-3 isoforms by MALDI QqTOF and ion trap mass spectrometry.** MALDI ion trap MS/MS spectra of unique tryptic peptides of 14-3-3ε (A), 14-3-3η (B).
(TIF)

Figure S8 **Identification of 14-3-3 isoforms by MALDI QqTOF and ion trap mass spectrometry.** MALDI ion trap MS/MS spectra of unique tryptic peptides of 14-3-3ζ (A), and 14-3-3θ/τ (B).
(TIF)
