# Correction: Phosphorylation-Dependent 14-3-3 Binding to LRRK2 Is Impaired by Common Mutations of Familial Parkinson's Disease

**DOI:** 10.1371/annotation/e66e7e90-9503-46b8-91f9-abe4d5056ba1

**Published:** 2011-07-21

**Authors:** Xianting Li, Qing Jun Wang, Nina Pan, Sangkyu Lee, Yingming Zhao, Brian T. Chait, Zhenyu Yue

Several grants are missing from the funding information. The complete funding information is:

"This work was supported by grants from the US National Institutes of Health/National Institute of Neurological Disorders and Stroke (NIH/NINDS) NS061152 (Z.Y.), NS060809 (Z.Y.), RNS055683A (Z.Y.), Michael J. Fox Foundation (Z.Y.), the US National Institutes of Health/National Center for Research Resources (NIH/NCRR) RR00862 (B.T.C.), RR022220 (B.T.C.), and 2P20 RR020171 (Q.J.W.). The funders had no role in study design, data collection and analysis, decision to publish, or preparation of the manuscript." 

