# Supplementary figures and images for: Phosphorylation-Dependent 14-3-3 Binding to LRRK2 Is Impaired by Common Mutations of Familial Parkinson's Disease
Source: PLoS One. 2011 Mar 1;6(3):e17153. doi: 10.1371/journal.pone.0017153 (PMC3046972; doi:10.1371/journal.pone.0017153)

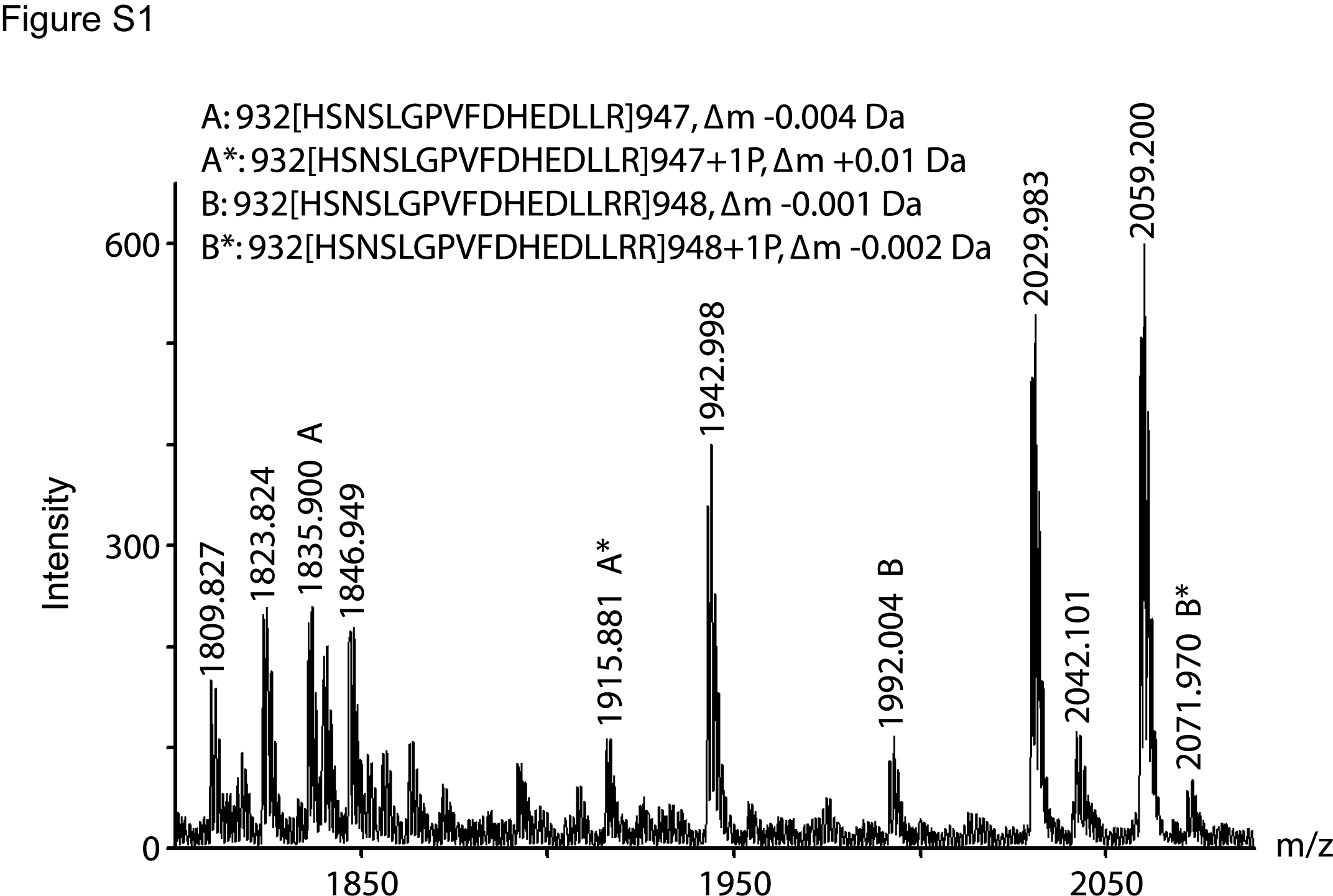

Supplement: Figure S1 — Identification of LRRK2 S935 phosphorylation by MALDI QqTOF and ion trap mass spectrometers. MALDI QqTOF mass spectrum of tryptic digested peptides extracted from the affinity purified mouse brain LRRK2 (band shown in Figure 5A). Two tryptic peptides containing the S935 phosphorylation site are labeled A* and B*. The corresponding unphosphorylated peptides are labeled A and B. (TIF) [file pone.0017153.s001.tif]

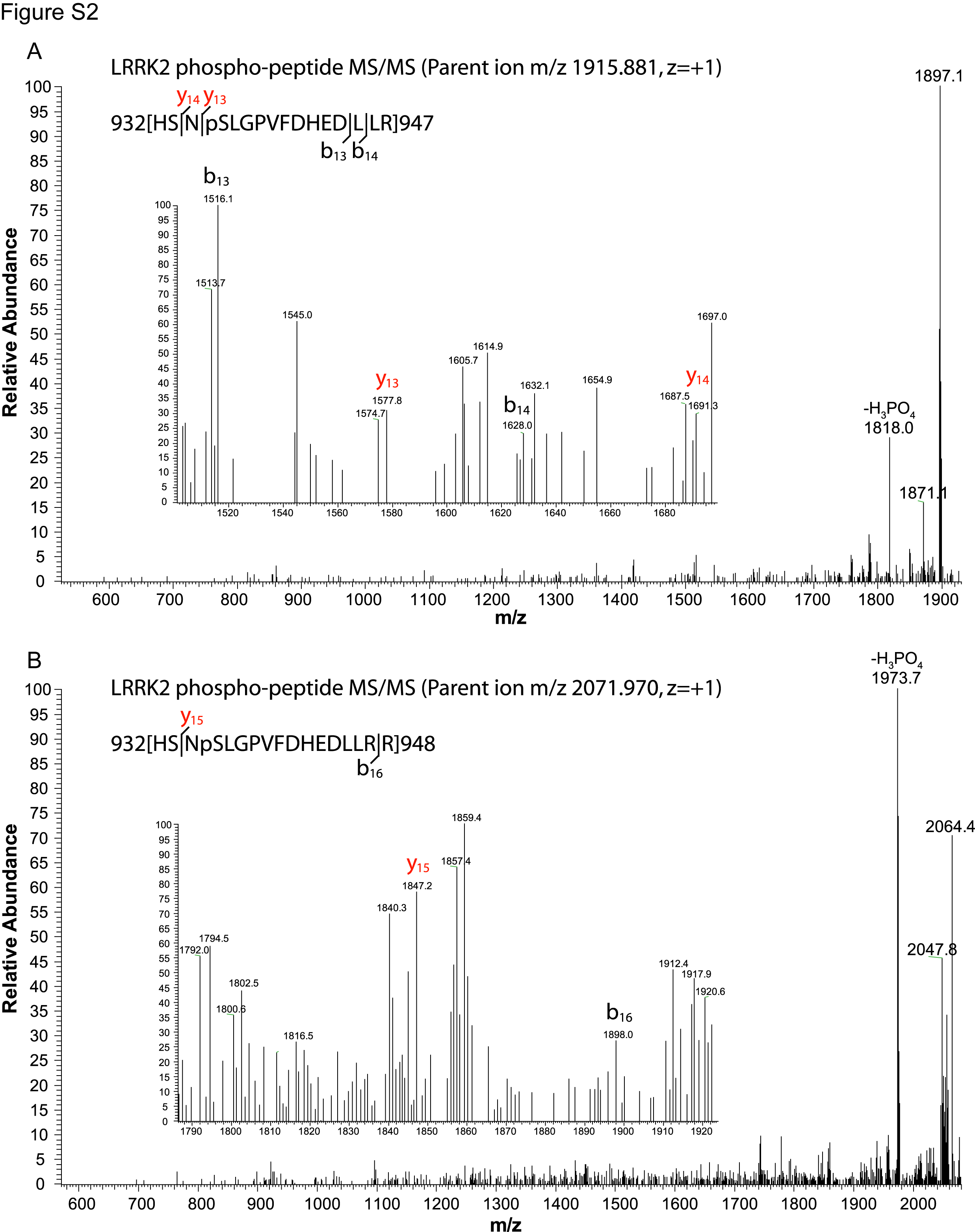

Supplement: Figure S2 — Identification of LRRK2 S935 phosphorylation by MALDI QqTOF and ion trap mass spectrometers. (A) MALDI ion trap MS/MS spectrum of the phosphorylated peptide HSNpSLGPVFDHEDLLR. (B) MALDI ion trap MS/MS spectrum of the phosphorylated peptide HSNpSLGPVFDHEDLLRR. Both MS/MS spectra show dominant peaks due to characteristic loss of H3PO4 (98 Da). For both (A) and (B), insets show zoomed-in regions of the spectra. Phosphorylated-S935-containing fragment ions are labeled in red. (TIF) [file pone.0017153.s002.tif]

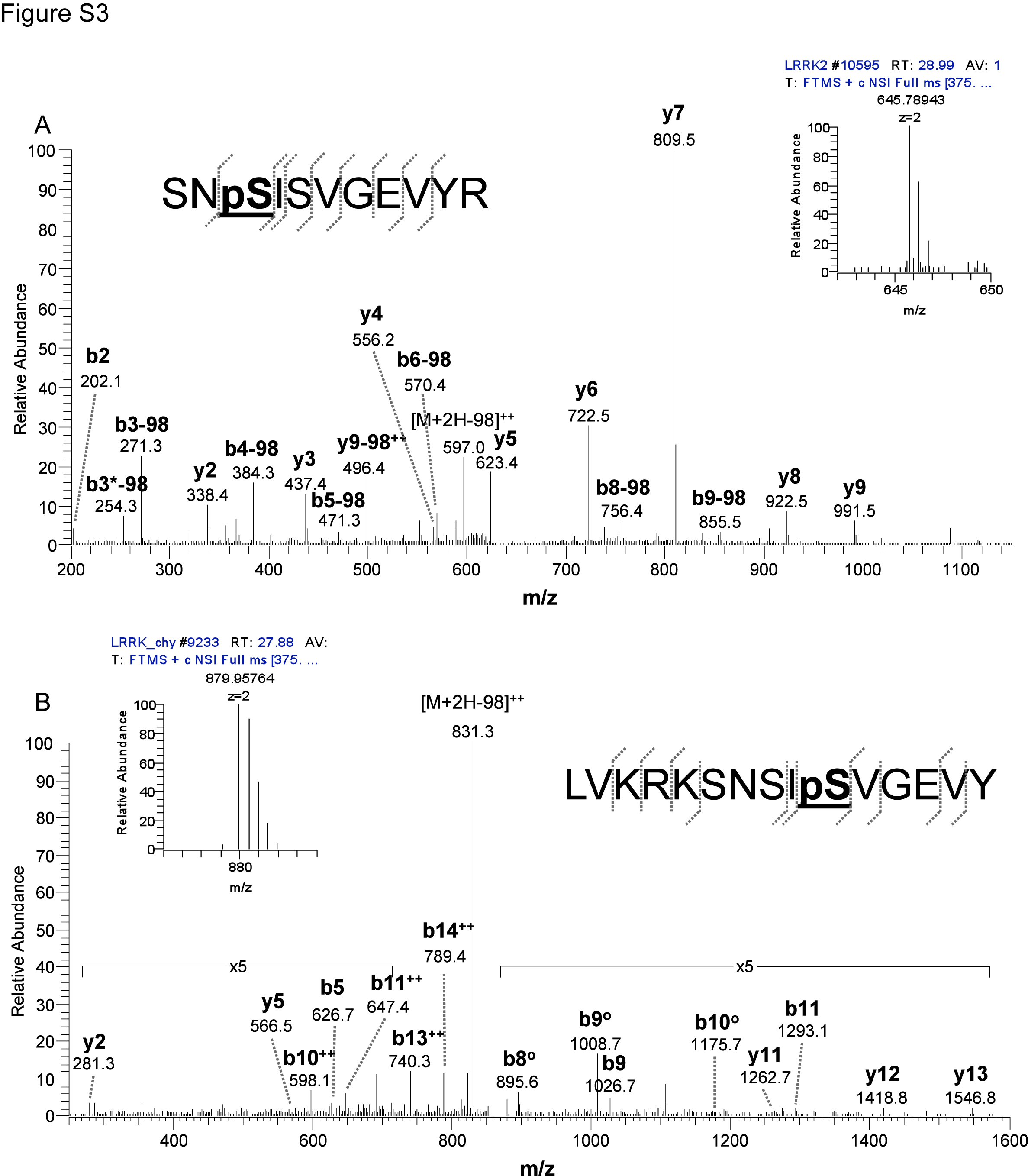

Supplement: Figure S3 — Analysis of phosphorylation sites in LRRK2 by nano-HPLC/velos LTQ Orbitrap mass spectrometer. MS/MS spectra of phosphorylated peptides at serine 910 (SNpSISVGEVYR) (A), serine 912 (LVKRKSNSIpSVGEVY) (B) in purified LRRK2 protein, respectively. In this study, we digested the purified LRRK2 protein in-gel using trypsin or chymotrypsin. The MS/MS data were analyzed by the Mascot algorithm to identify the protein and its posttranslational modifications. The candidate peptides bearing the serine phosphorylation were further examined manually as previously described [45]. The phosphorylated peptide can be identified by a mass shift of 79.96633 Da at serine/threonine/tyrosine residues. (TIF) [file pone.0017153.s003.tif]

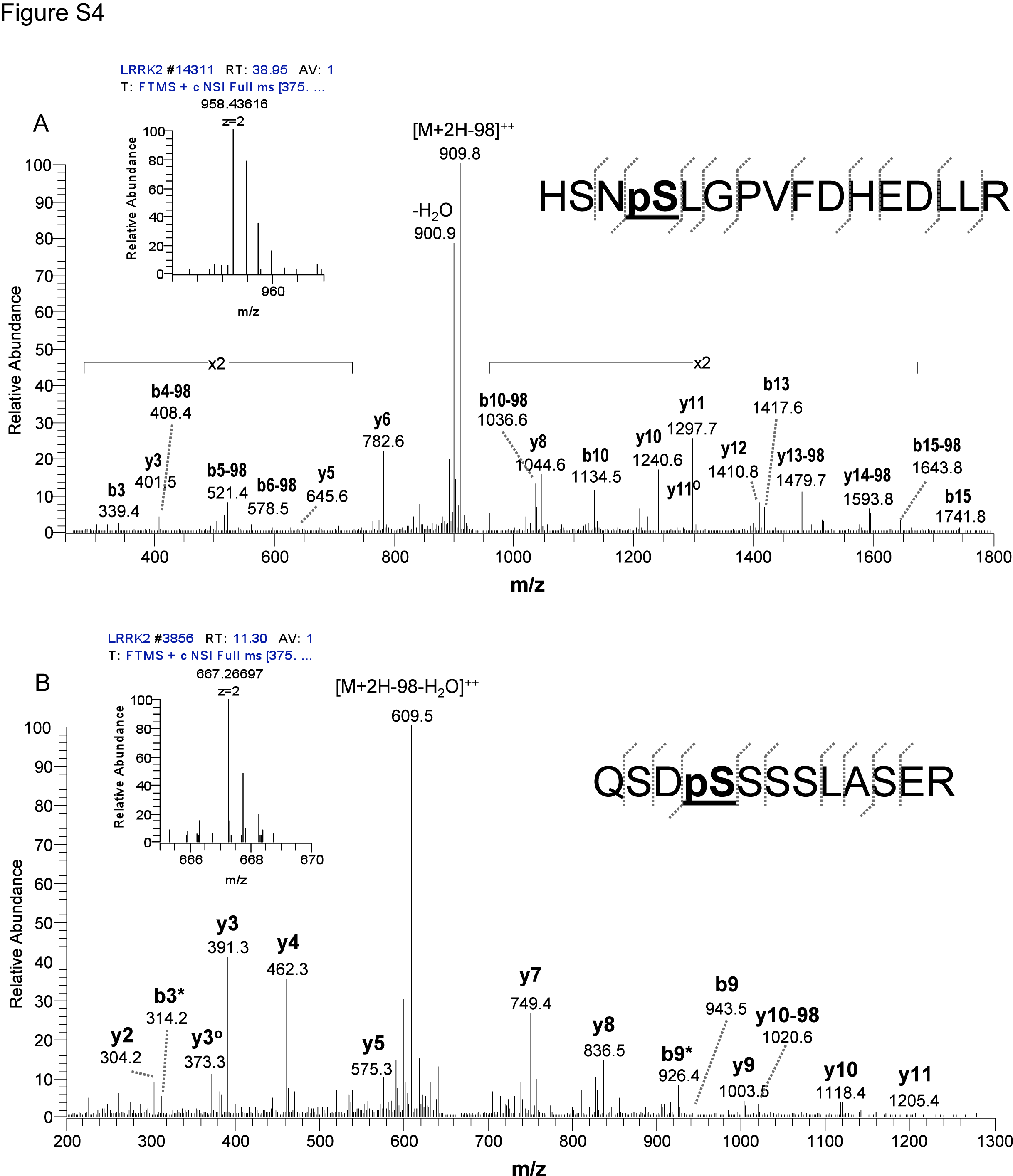

Supplement: Figure S4 — Analysis of phosphorylation sites in LRRK2 by nano-HPLC/velos LTQ Orbitrap mass spectrometer. MS/MS spectra of phosphorylated peptides at serine 935 (HSNpSLGPVFDHEDLLR) (A), and serine 973 (QSDpSSSSLASER) (B) in purified LRRK2 protein, respectively. In this study, we digested the purified LRRK2 protein in-gel using trypsin or chymotrypsin. The MS/MS data were analyzed by the Mascot algorithm to identify the protein and its posttranslational modifications. The candidate peptides bearing the serine phosphorylation were further examined manually as previously described [45]. The phosphorylated peptide can be identified by a mass shift of 79.96633 Da at serine/threonine/tyrosine residues. (TIF) [file pone.0017153.s004.tif]

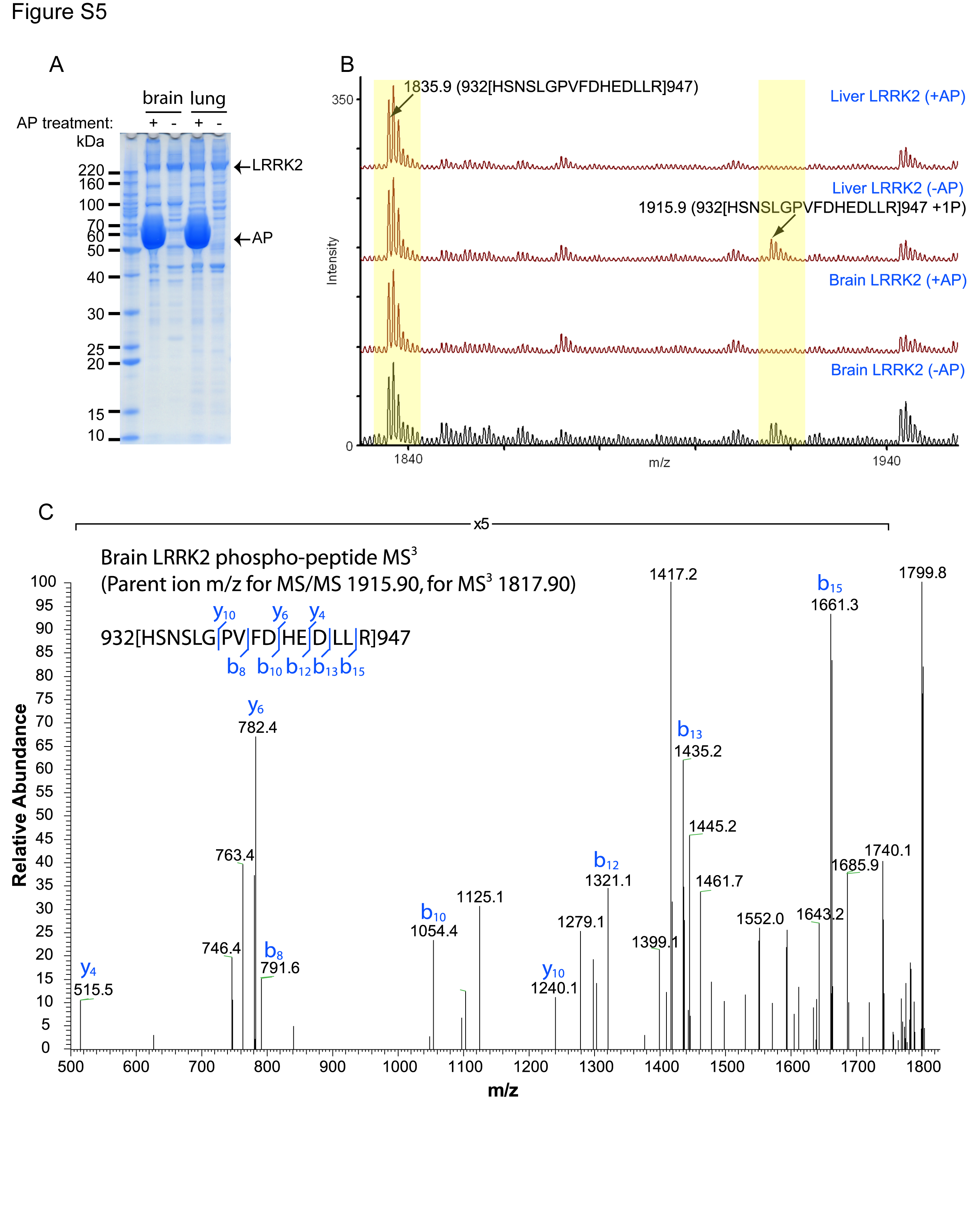

Supplement: Figure S5 — Confirmation of LRRK2 S935 phosphorylation by a combination of alkaline phosphatase treatment and mass spectrometry. (A) Coomassie blue stain gel showing affinity purified FLAG-LRRK2 and its interacting proteins from FLAG-LRRK2 BAC transgenic mouse brain and lung, with and without alkaline phosphatase (AP) treatment. LRRK2 and AP bands are labeled by arrows. AP treatment was performed by incubating anti-FLAG immunoprecipitation eluent in AP (Roche, 2 U/µL) at 37°C for 1 h. (B) MALDI QqTOF mass spectra of LRRK2 tryptic digested peptides extracted from the gel bands shown in (A). The monoisotopic peaks of the unphosphorylated and phosphorylated LRRK2 tryptic peptide 932[HSNSLGPVFDHEDLLR]947 are highlighted in yellow. (C) MS3 tandem mass spectrum confirming phosphorylated LRRK2 tryptic peptide 932[HSNpSLGPVFDHEDLLR]947, using a MALDI ion trap mass spectrometer. (TIF) [file pone.0017153.s005.tif]

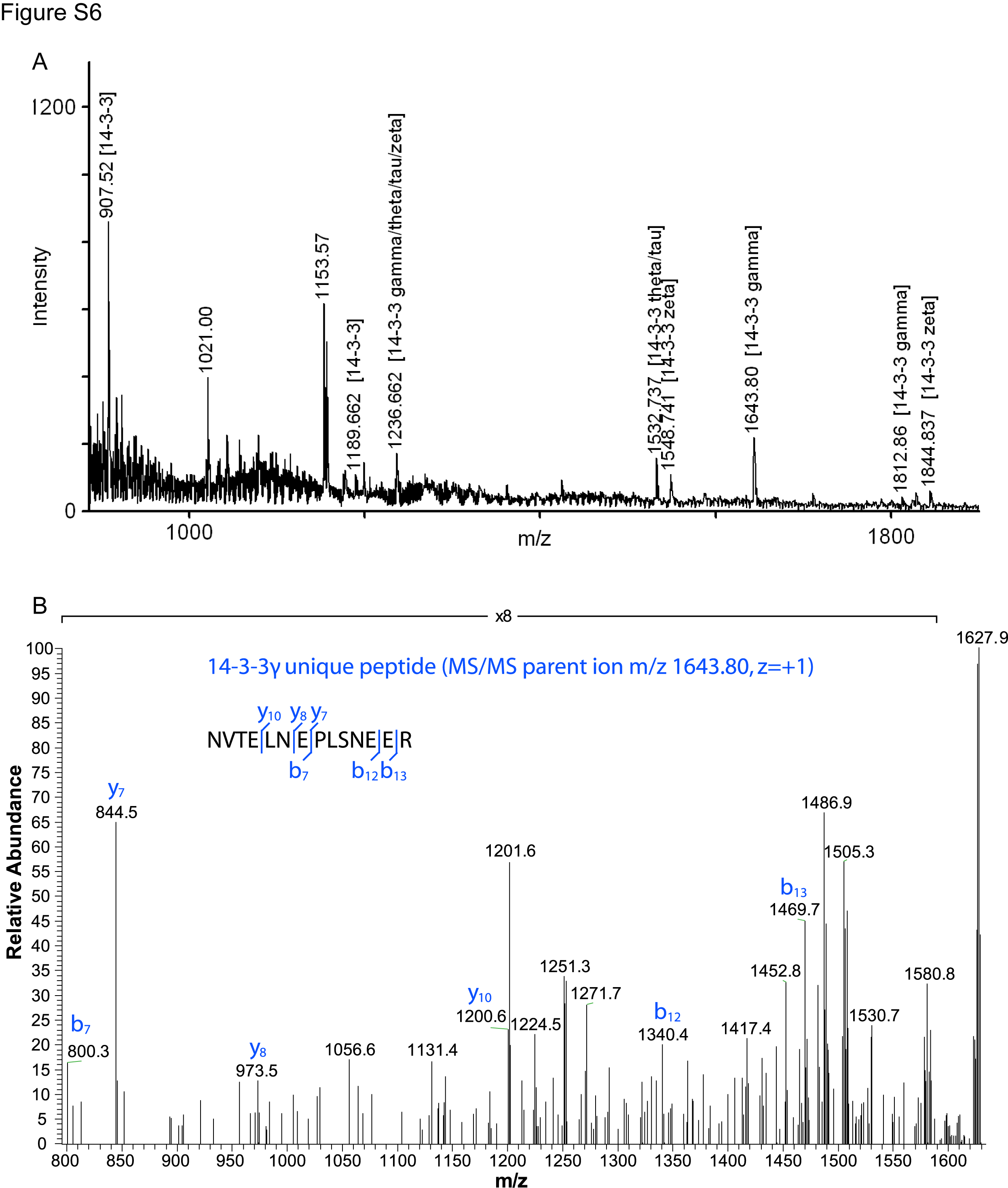

Supplement: Figure S6 — Identification of 14-3-3 isoforms by MALDI QqTOF and ion trap mass spectrometry. (A) MALDI QqTOF mass spectrum of tryptic digested peptides extracted from the affinity purified mouse brain 14-3-3 (band shown in Figure 5A). (B) MALDI ion trap MS/MS spectra of unique tryptic peptides of 14-3-3γ. (TIF) [file pone.0017153.s006.tif]

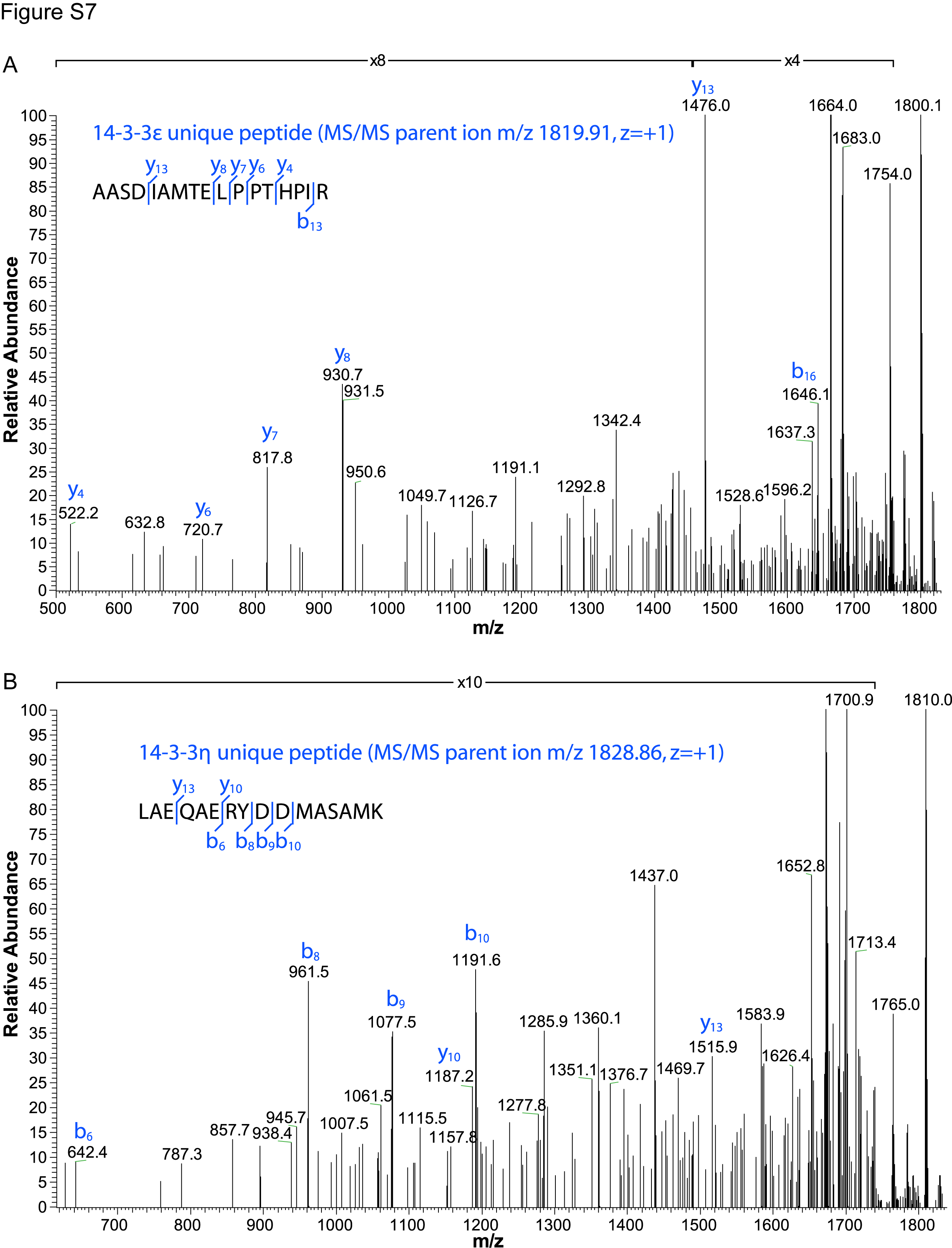

Supplement: Figure S7 — Identification of 14-3-3 isoforms by MALDI QqTOF and ion trap mass spectrometry. MALDI ion trap MS/MS spectra of unique tryptic peptides of 14-3-3ε (A), 14-3-3η (B). (TIF) [file pone.0017153.s007.tif]

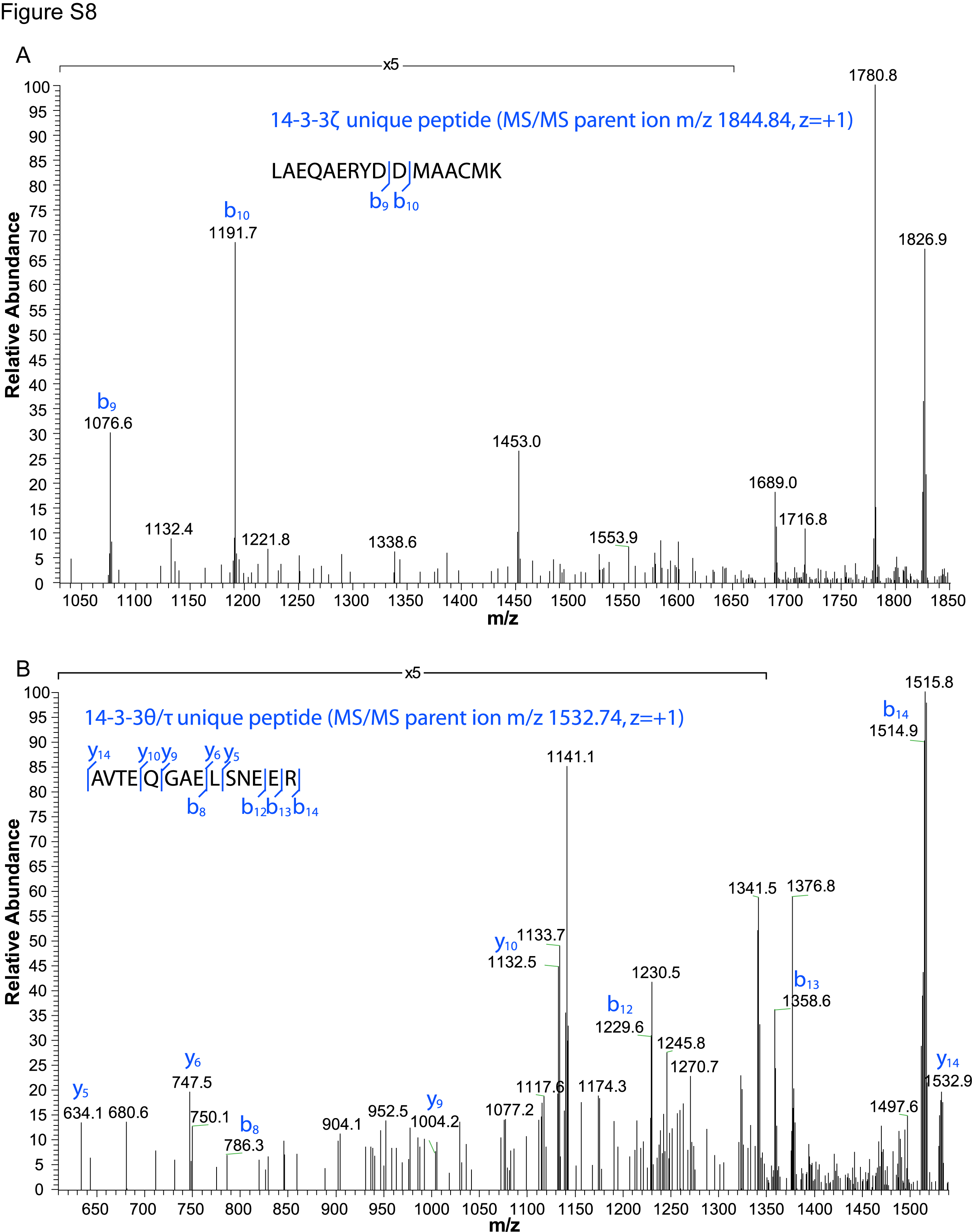

Supplement: Figure S8 — Identification of 14-3-3 isoforms by MALDI QqTOF and ion trap mass spectrometry. MALDI ion trap MS/MS spectra of unique tryptic peptides of 14-3-3ζ (A), and 14-3-3θ/τ (B). (TIF) [file pone.0017153.s008.tif]
